# Spatially resolved sampling for untargeted metabolomics: A new tool for salivomics

**DOI:** 10.1016/j.isci.2021.102768

**Published:** 2021-06-24

**Authors:** Alessio Ciurli, Maximiliam Liebl, Rico.J.E. Derks, Jacques J.C. Neefjes, Martin Giera

**Affiliations:** 1 Oncode Institute and Department of Cell and Chemical Biology, Leiden University Medical Center, Leiden 2333 ZA, the Netherlands; 2 Center for Proteomics and Metabolomics, Leiden University Medical Center, Leiden 2333 ZA, the Netherlands

**Keywords:** Spatially resolved sampling, Untargeted metabolomics, Liquid chromatography and tandem mass spectrometry, Saliva, Salivomics

## Abstract

Saliva is a complex bodily fluid composed of metabolites secreted by major and minor glands, as well as by-products of host oral cells, oral bacteria, gingival crevicular fluid, and exogenous compounds. Major salivary glands include the paired parotid, submandibular, and sublingual glands. The secreted fluids of the salivary glands vary in composition, flow rate, site of release, and clearance suggesting that different types of saliva fulfill different functions and therefore can provide unique biological information. Consequently, for the comprehension of the functionality of the salivary components, spatially resolved investigations are warranted. To understand and comprehensively map the highly heterogeneous environment of the oral cavity, advanced spatial sampling techniques for metabolomics analysis are needed. Here, we present a systematic evaluation of collection devices for spatially resolved sampling aimed at untargeted metabolomics and propose a comprehensive and reproducible collection and analysis protocol for the spatially resolved analysis of the human oral metabolome.

## Highlights

• Systematic evaluation of collection devices for untargeted metabolomics of saliva
• Spatially resolved sampling of saliva in the human oral cavity
• Enabling location-specific oral metabolomics

## Graphical abstract

**Figure:**
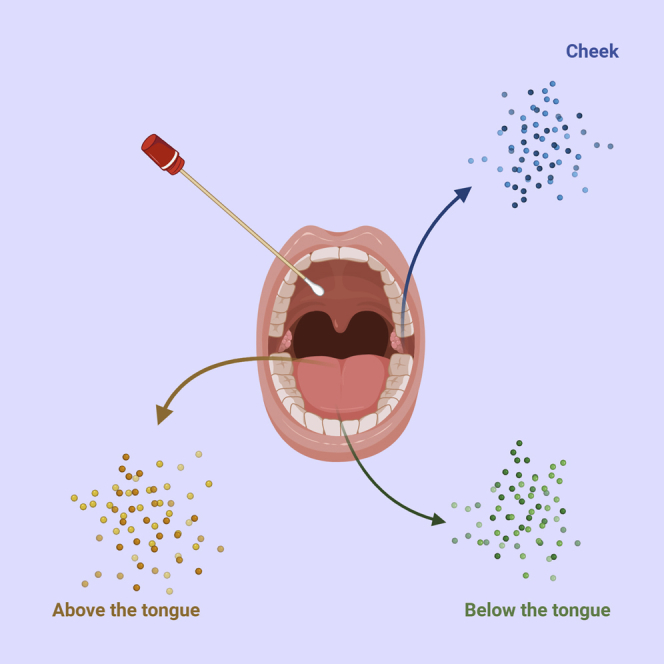

## Teaser

Body substance sample; Metabolomics; Omics

## Introduction

Owing to its accessibility and facile (non-invasive) collection, saliva has been proposed as a future diagnostic fluid (Granger et al., 2007). Saliva is undoubtedly a valuable source of molecular information, reflective of the oral cavity. Moreover, in many cases, metabolic changes observed in saliva are paralleled by systemic metabolic alterations (Paine and Snider, 2020; Zerihun et al., 2015). Salivary samples may be analyzed for levels of endogenous, therapeutic, or recreationally used substances; hormonal status; immunologic status; neurologic status; and nutritional metabolic influences (Humphrey and Williamson, 2001). Saliva is a complex bodily fluid composed of components mainly produced by major and minor glands, and also by-products of host oral cells, oral bacteria, gingival crevicular fluid (serum-derived filtrate fluid), and exogenous compounds as, for example, residual foods or oral hygiene products (Gardner et al., 2020). Major salivary glands include the paired parotid, submandibular, and sublingual glands. Parotid saliva is released on the cheeks through the Stensen duct, whereas submandibular and sublingual saliva are both released on the mouth floor through the Wharton and Bartholin ducts, respectively. Minor glands producing saliva are found in the lower lip, tongue, palate, cheeks, and pharynx (Humphrey and Williamson, 2001). The secreted fluids of the salivary glands vary significantly. Serous-type fluids are released from the parotid gland, mucous-type from minor glands, and mixed-type secretions from sublingual and submandibular glands (Humphrey and Williamson, 2001). These gland-specific fluids display variable compositions (Veerman et al., 1996) suggesting that different types of saliva fulfill different functions and consequently can provide unique biological information. Several techniques may be used to collect whole saliva (e.g., whole mouth saliva [WS] collection, passive drool, or by using absorbent swabs) (Khurshid et al., 2016; Navazesh, 1993). However, gland-specific saliva collection might be a better choice for diagnostic purposes. Yet, gland-specific saliva collection generally requires complex procedures, highly skilled staff, even risking injuries (e.g., intraductal cannulation) (Navazesh, 1993) or the need for personalized devices (Veerman et al., 1996). Other techniques such as the Lashley cup collector and suction may solve some of the above-mentioned issues, but their use is restricted to a single gland (Navazesh, 1993; Roesink and Terhaard, 2002). Moreover, single gland saliva collection is limited to the major glands and does not reflect the contribution of numerous minor glands. As a result of site-specific release and clearance, salivary components are unlikely to be uniformly distributed. As suggested by Dawes and MacPherson, even during gum chewing saliva still provided a series of distinctly site-specific environments (Dawes and Macpherson, 1993). Thus, for comprehending the functionality of all salivary components, saliva should be investigated in a thorough, spatially resolved fashion. Although previous reports have mainly focused on WS (Ovchinnikov et al., 2014; Lim et al., 2018), we believe it is paramount to understand and comprehensively map the highly heterogeneous biochemical environment of the oral cavity using advanced spatial sampling techniques for metabolomics analysis. Of importance, spatially resolved saliva (SRS) sampling might open new possibilities in mapping and understanding the local biological environment through (1) spatial correlation of metabolites and oral microbiota, enabling integrated multi-omics studies, and/or (2) spatially linking metabolic changes with oral pathological conditions, providing spatial metabolic information at or near local disease areas. Important fields for such investigations include periodontal disease as well as head and neck cancer (Song et al., 2020) where spatially resolved sampling could present a simple and robust alternative to desorption-ionization mass spectrometry (D'hue et al., 2018), leading to entirely novel insights into local environments. Here, we present a systematic evaluation of collection devices for spatially resolved saliva sampling aimed at untargeted metabolomics and propose a solid collection and analysis protocol for the spatially resolved analysis of the human oral metabolome.

## Results

### Volume recovery

**Figure 1:**
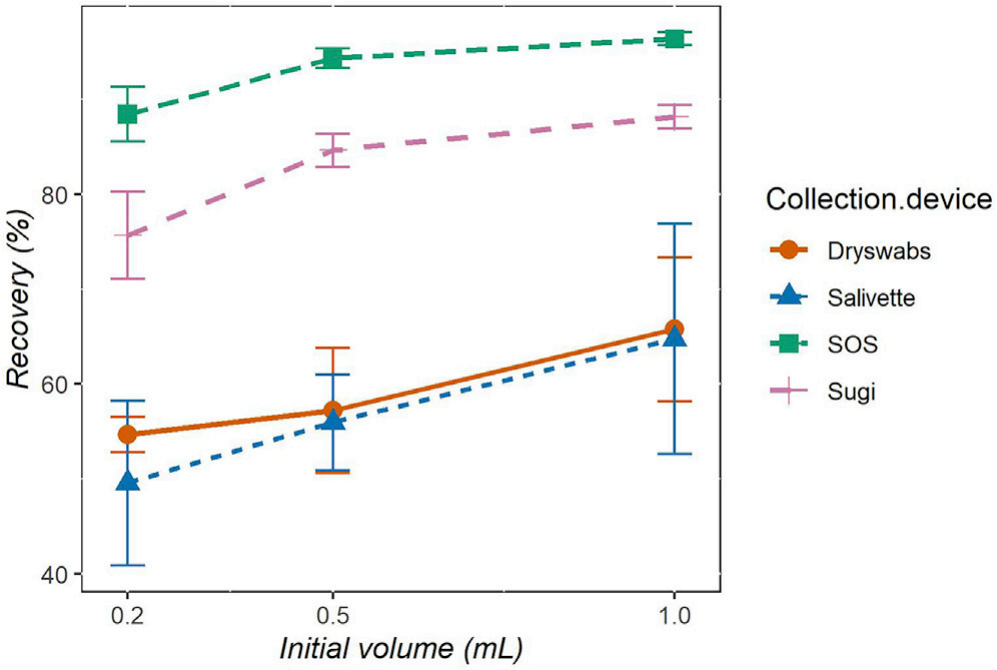
Saliva volume recovery The percentage of saliva volume recoveries (y axis) are displayed per collection device in relation to the initial volume used (x axis). Data are represented as mean ± SD (n = 5).

A high recovery of biological fluids is an essential property for every collection device. Volume recovery is a predictor of the smallest volume collectable as well as an indication of the fraction of biological sample retained by the device. All investigated devices showed increasing volume recovery with increasing initial test volumes (Figure 1). However, Dryswab and Salivette showed poor recovery and reproducibility across all tested initial volumes (max Rec % = 65.8% and 64.8% for Dryswab and Salivette, respectively). Sugi displayed a better performance by maintaining a good volume recovery across all volumes tested (Rec % = 88.1%, 84.6%, and 75.6%, for 1, 0.5, and 0.2 mL, respectively) as well as a good reproducibility (relative standard deviation (RSD) = 1.4%, 2.1%, and 6.1% for 1, 0.5, and 0.2 mL, respectively). Finally, SOS showed the best performance among the devices tested, with the highest recovery (Rec % = 96.3%, 94.3%, and 88.4% for 1, 0.5, and 0.2 mL, respectively) and the highest reproducibility (RSD = 0.7%, 1.1%, and 3.2% for 1, 0.5, and 0.2 mL, respectively). Taken together, Sugi and SOS display a satisfactory volume recovery and reproducibility.

### Background contamination

**Figure 2:**
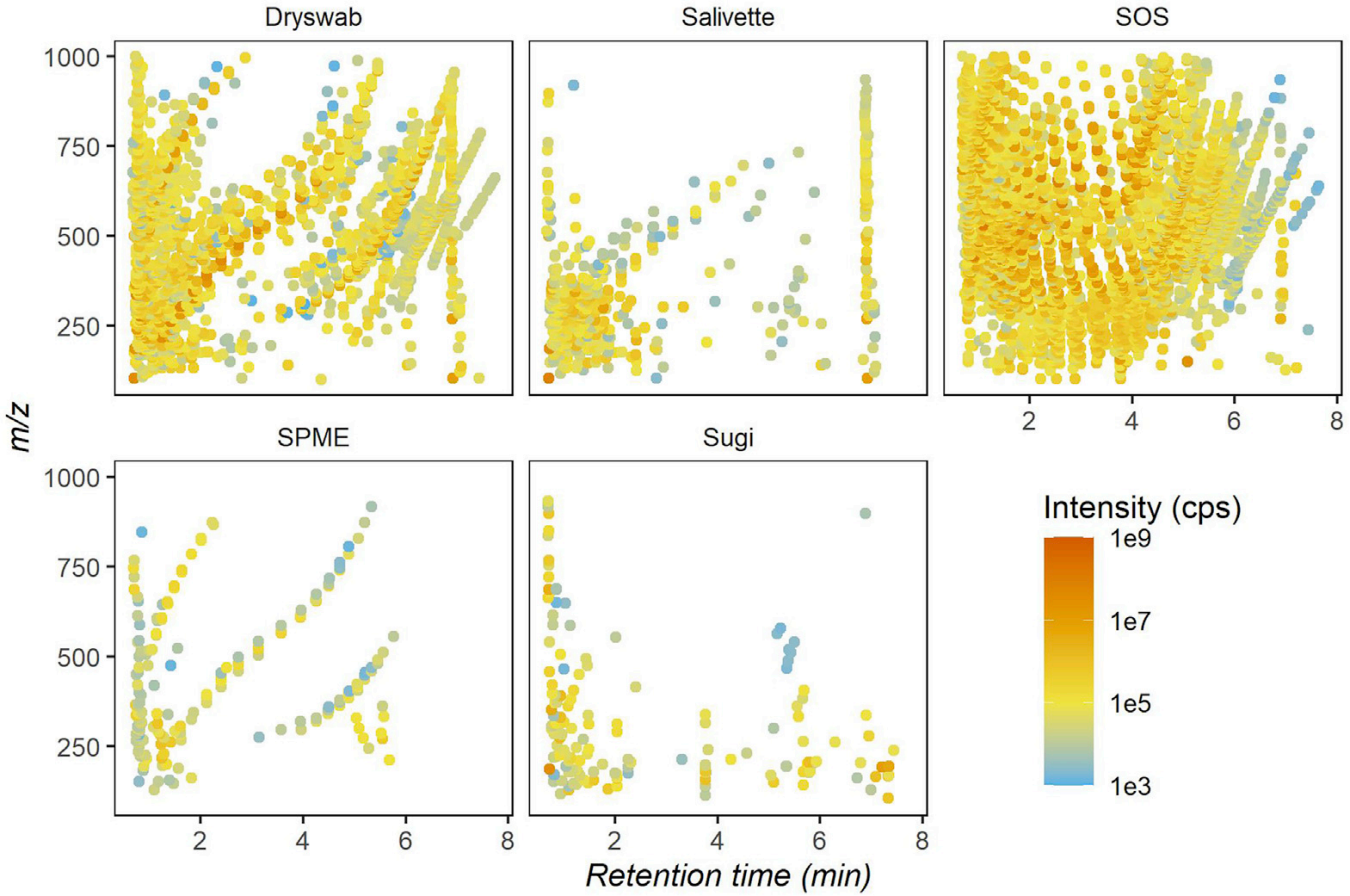
Background contamination by ESI+ mode The sequence of 2D plots displays the contaminants released per collection device. Contaminants are displayed based on their mass to charge (y axis) and retention time (x axis) coordinates and colored based on the average of signal intensities as shown in the legend. Data are represented as mean (n = 5). For result obtained in ESI- mode see Figure S1.

The quality of mass spectral data is often affected by background contamination, which might result in lower sensitivity often caused by matrix effects. Particularly in the case of untargeted analysis, high background noise may significantly influence the obtained results and data interpretation. Hence, as a next step in our investigation, we tested the collection devices for their contribution to background contamination during LC-MS analysis. Results from LC-MS background contamination analysis in ESI+ mode (Figure 2) showed a high number of contaminants characterized by intense LC-MS signals mainly found for Dryswab and SOS. The type of contaminations is likely of polymeric nature as can be argued by constant *m/z* and retention time spacing. Our results show that both devices suffer from high chemical background noise during LC-MS analysis. Salivette and SPME overall displayed a much lower number and intensity of background LC-MS signals when compared with Dryswab and SOS. Finally, Sugi displayed the cleanest profile among the tested swab devices without traces of polymeric contaminants. The ESI- results (Figure S1) display a similar trend for all tested devices.

Owing to both the high chemical background and the poor volume recovery capacities, we excluded the Dryswab device from further evaluation at this point.

### Molecular feature recovery

**Figure 3:**
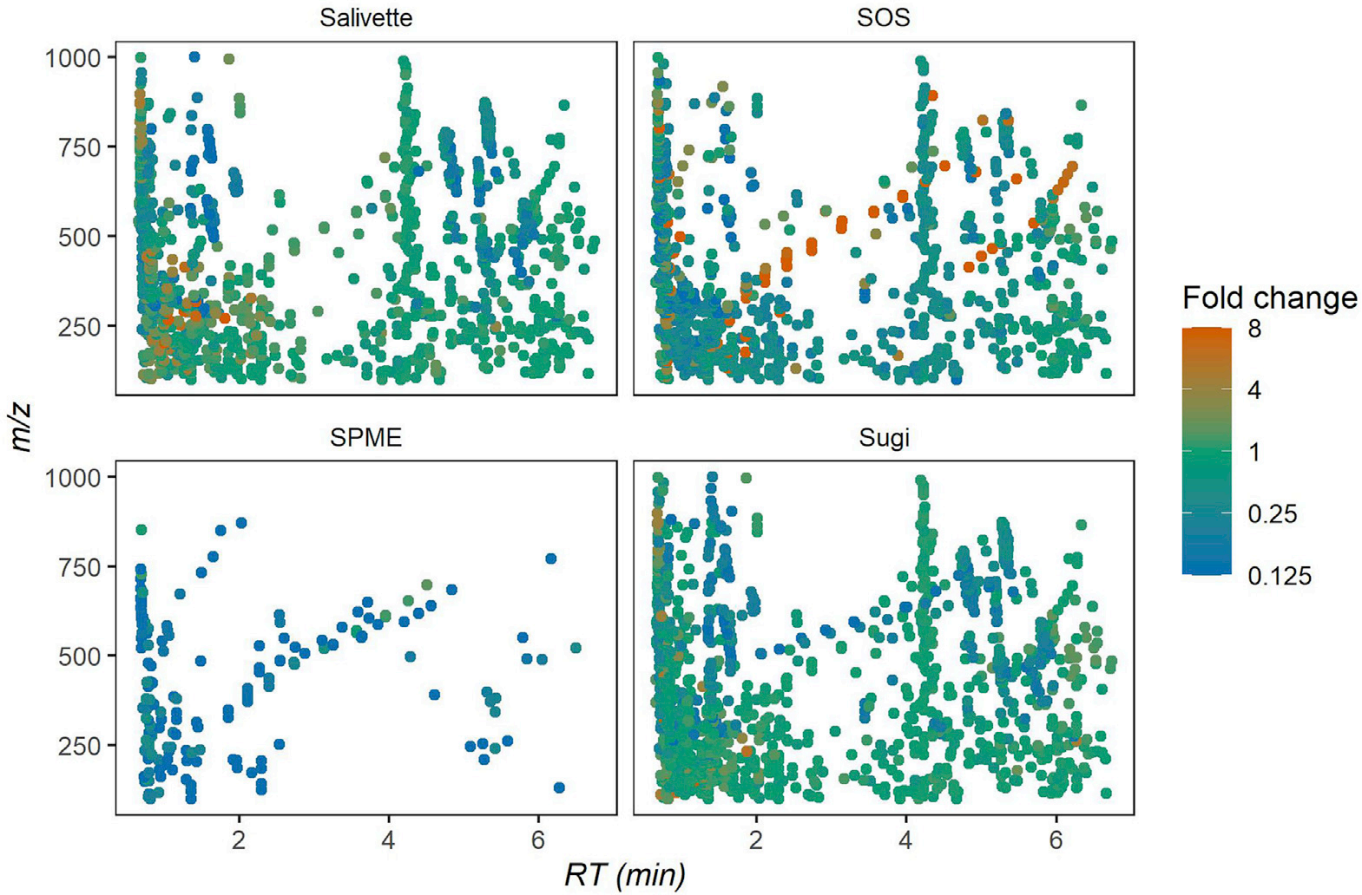
Molecular features recovery by ESI+ mode The sequence of 2D plots displays the molecular features recovered per collection device. Molecular features are displayed based on their mass to charge (y axis) and retention time (x axis) coordinates. Color bar indicates the fold change of the average signal intensity of sampled saliva over the average of non-sampled saliva. Data are represented as mean (n = 5). For result obtained in ESI- mode see Figure S2.

**Figure 4:**
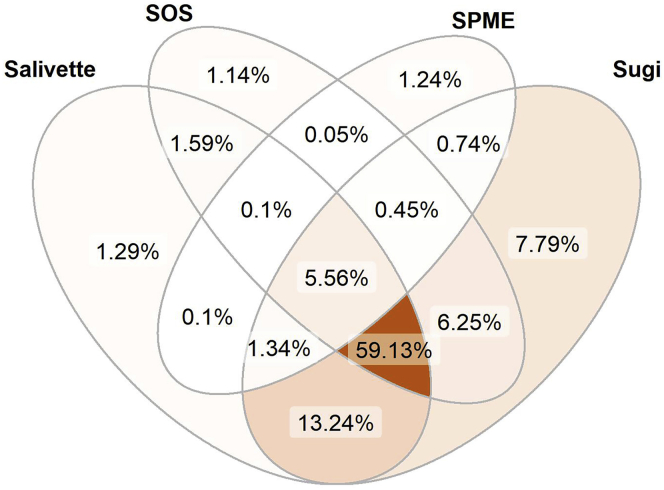
Overlap of features captured by ESI+ mode The Venn diagram displays the percentage of detected features by each device (n = 5) and the overlaps among the devices. Venn diagram sections are colored based on the percentage of features belonging to each section. For result obtained in ESI- mode see Figure S3.

**Figure 5:**
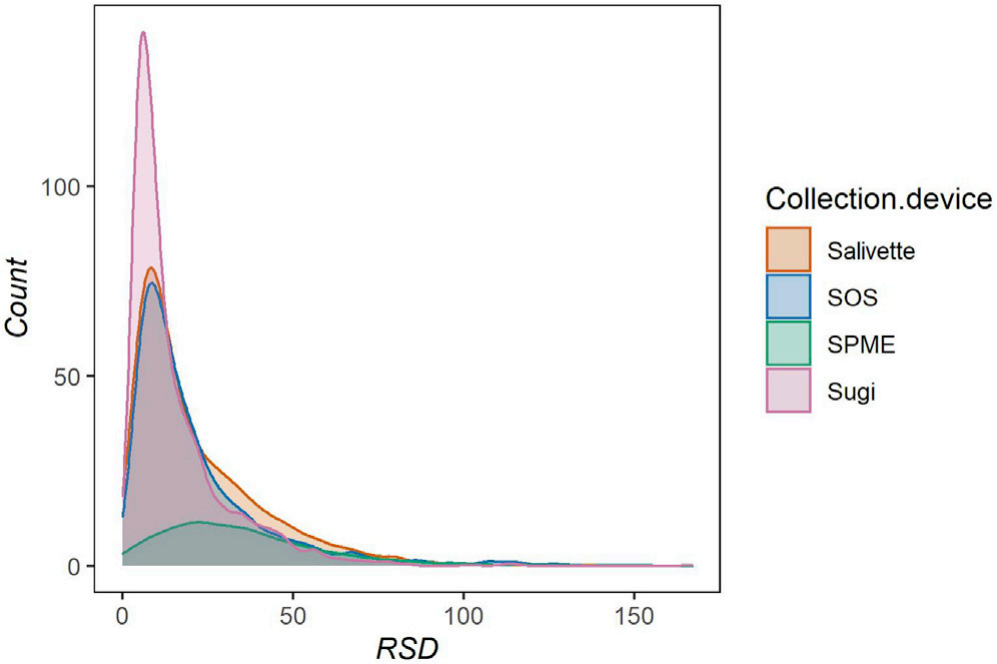
Reproducibility by ESI+ mode The density plot displays the distribution of RSDs belonging to the molecular features recovered by each device (n = 5), as reported in the legend. For result obtained in ESI- mode see Figure S4.

The here-presented approach is aimed to provide a sampling method for untargeted metabolomics. In turn, we wanted to draw a comprehensive comparison of detectable molecular features in non-sampled compared with sampled saliva. Therefore, each device was evaluated for metabolite recovery and reproducibility. On analysis of non-sampled saliva with our LC-MS platform, 2,256 molecular features were detected in the ESI+ mode and 1,118 in the ESI- mode. The results using the different collection devices in the ESI+ mode display very limited recoveries for most features using SPME, 75.4% of the features were missing. The other three devices showed progressive improvement in the following order: SOS, Salivette, and Sugi. SOS and Salivette displayed a significant number of missing features being 34.2% and 22.8%, respectively. Sugi outperformed all other devices tested by showing a marginal portion of missing features (11%) and considering the high number of features characterized by a high degree of fidelity (fold changes ~1) (Figure 3). Although SOS and Salivette were also able to recover most of the features, the majority of these were recovered by Sugi as well, which covered almost the entire pool of salivary features recovered (Figure 4). Finally, the reproducibility of features recovery per device was investigated by comparing the RSD distribution (Figure 5). The observed ESI- results displayed a similar trend (Figures S2–S4) with a significantly improved performance of SOS when comparing ESI- and ESI+ modes. In summary, Sugi outperformed all tested devices for matrix fidelity, coverage, and reproducibility when investigating molecular feature recoveries in both ESI- and ESI+ modes.

### Spatially resolved sampling

**Figure 6:**
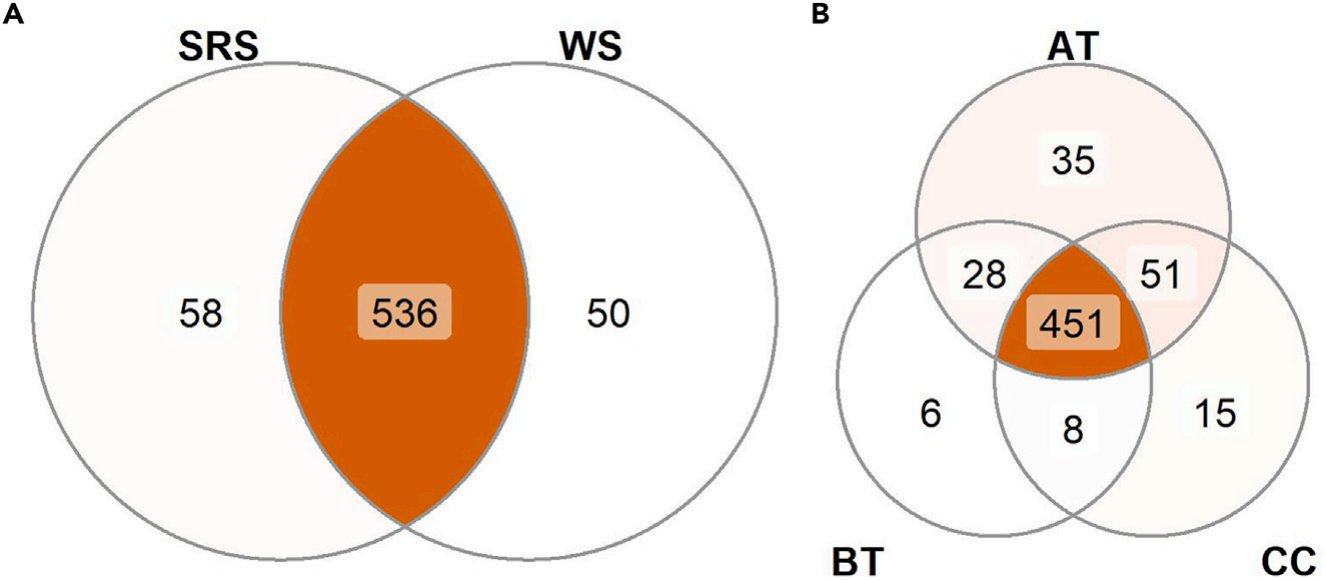
Sampling method comparison (A) The Venn diagrams display the common and unique molecular features per sampling method, whole saliva (WS) and spatially resolved saliva (SRS) (WS n = 9, SRS n = 27), and (B) per location in the case of the spatially resolved sampling method, above the tongue (AT), below the tongue (BT), and cheeks (CC) (AT n = 8, BT n = 8, and CC n = 11).

After having performed a systematic evaluation of collection devices, the best performing device for untargeted profiling of saliva (Sugi) was used to carry out spatially resolved sampling of the oral cavity with the purpose of testing the advantage of SRS sampling over WS collection. For our test case, four different locations were sampled in the oral cavity of healthy volunteers (n = 9): above the tongue (AT), below the tongue (BT), and left cheek and right cheek (CC). The vast majority of detected molecular features (#536) were shared between WS and SRS samples. However, spatially resolved sampling did result in 58 unique features when compared with WS analysis. We also detected 50 unique molecular features in WS (Figure 6A). In addition, spatially resolved sampling was able to reveal uniquely detected features characteristic for the sampled oral location; 35 uniquely detected features were found for AT, 15 were characteristic for CC, and 6 were characteristic for BT (Figure 6B).

**Figure 7:**
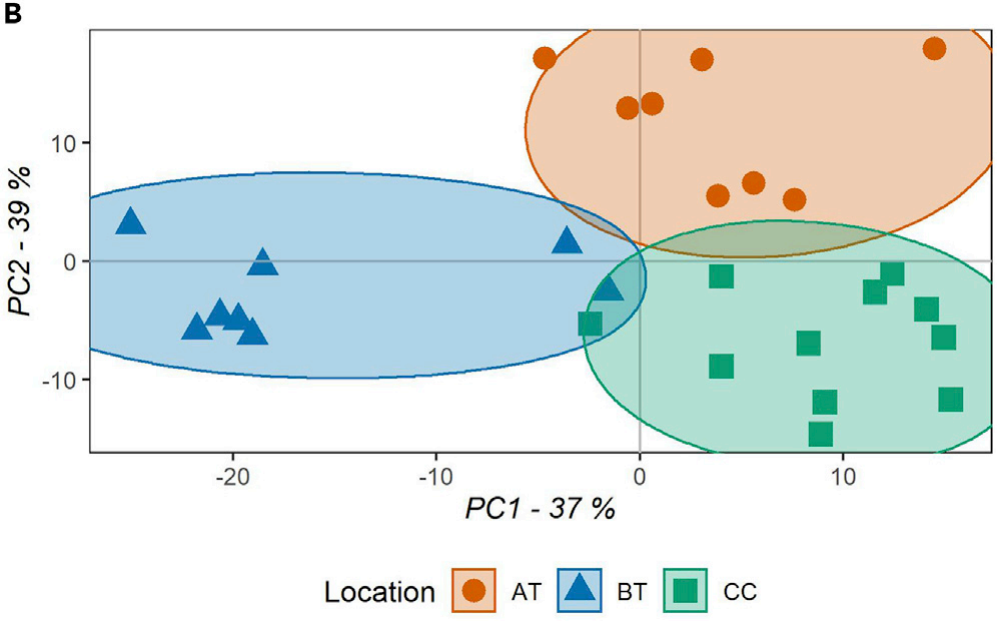
PLSDA score plot The score plot displays the sample distribution for principal component one (x axis) and two (y axis), on the axis label the explained variance for each principal component is reported. Samples are colored based on the location of collection (AT, above the tongue; BT, below the tongue; CC, cheeks), as reported in the legend (AT n = 8, BT n = 8, and CC n = 11).

**Figure 8:**
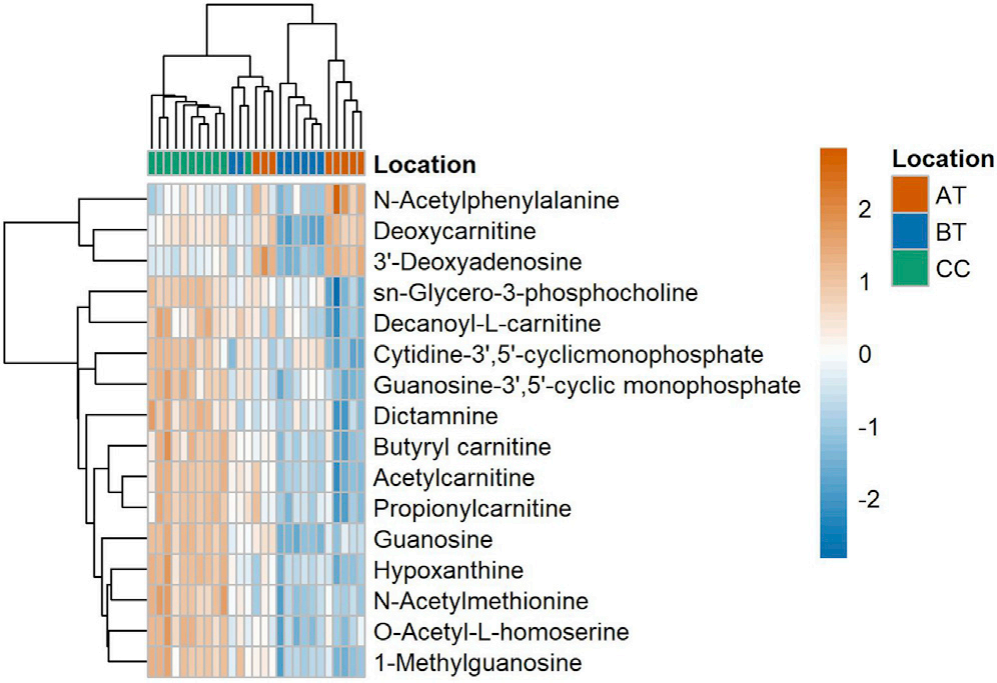
Location characteristic heatmap The heatmap and associated hierarchical cluster of the samples were obtained with the top VIPs (>1.5) among the identified metabolites. The heatmap was generated using the spatially resolved samples and labeled based on the location of collection (AT, above the tongue, BT, below the tongue; and CC, cheeks). Data are centered, UV scaled, and log10 transformed (AT n = 8, BT n = 8, and CC n = 11).

After common and unique feature detection, their distribution within the oral cavity was investigated using PLSDA and cluster analysis. Location comparison of SRS samples led to three clusters (Figure 7). A very small overlap among the clusters might be caused by the inter-subject variability. Besides the strict inclusion criteria limiting gender variability (only male volunteers), age influence (18–45 years old) and influence of circadian rhythm (sampling time 08.30 a.m.–10.30 a.m.), other factors like diet, smoking habits, sleep pattern, and degree of physical activity might play a role. Finally, using VIP scores generated by the PLSDA, the metabolite distribution of the highest VIPs (>1.5) among the identified metabolites is displayed as a hierarchical cluster (Figure 8). Diagnostics plots and a heatmap generated with all detected metabolites can be found in Figures S5 and S6. All detected features (#667), retention time, *m/z*, MS2 spectra, and identity (#88) (in case of library match) are reported in Data S1. In summary, using the human oral cavity as a test case, spatially resolved sampling allowed us to uniquely detect metabolites belonging to specific oral locations, as well as to map the oral distribution of common metabolites across the oral cavity.

## Discussion

Here we report that a careful and systematic evaluation of collection devices for saliva analysis by untargeted metabolomics is a critical pre-requisite for a robust sampling protocol. We detected vast differences in performance between all tested devices. The Dryswab device showed not only a very limited volume recovery (65.8% for 1 mL) but also a significant degree of background contamination. Consequently, we excluded this device early on in our investigation. SPME was previously described for saliva sampling (Bessonneau et al., 2013, 2015) and successfully applied to monitor prohibited substances (Hall et al., 1998; Souza et al., 2011). Also, in our hands we obtained promising results from the contamination test in both ESI+ and ESI-. However, the low degree of contamination coincided with a very poor recovery resulting in almost 75.4% loss of features when metabolite recovery was studied. Possibly, the fact that we used a very short sampling time of only 2 min combined with the use of an SPME needle rather than a thin-film SPME device (Bessonneau et al., 2015) may have contributed. The short sampling time was for practical reasons; volunteer appreciation and also the time of clinicians as well as ease of sampling and commercial availability of the device. The Salivette device displayed the lowest recovery and the highest spread of recovery for all volumes tested. This might be explained by a shorter centrifugation step for volume recovery when compared with the other devices. However, the extraction procedures used followed instructions provided by the manufacturer. The background contaminant test for the Salivette device showed a high concentration of contaminants in the early-retention-time and low-mass-to-charge zone (<3 min and < *m/z* 400). The SOS device showed the best performance in the volume recovery test, where the lowest rate of recovered volume equaled 88.4% with an RSD of only 3.2% when 0.2 mL was available for absorption. However, the background contamination test displayed severe contamination for analysis in ESI+ and ESI- modes. Ultimately, this result made a further consideration of the device impossible for untargeted metabolomics analysis. Of note, the SOS device has been validated and used for the analyses of salivary analytes (e.g., cortisol, melatonin) (de Bernardo et al., 2018; Tapia et al., 2018), highlighting the significantly different analytical needs of targeted and untargeted LC-MS approaches. Finally, the Sugi device was characterized as the most robust and constant collection device tested with a reproducible volume recovery for all initial volumes tested (Rec % = 75.6%, with RSD = 6.1% for 0.2 mL), the lowest level of contaminants in both ESI+ and ESI- modes, as well as a significant and reproducible features recovery. Ultimately, the Sugi device was used to perform spatially resolved sampling and results were compared with the WS collection. An equal number of molecular features was detected for both the SRS and WS collection (Figure 5). Although unique metabolites observed collecting WS might reflect strong analyte absorbance to the Sugi swab, spatially resolved sampling might result in a simplified matrix and thereby reduce matrix effects and thereby increase detection of specific metabolites. Ultimately, as a relevant test case for the here-presented spatial sampling protocol, we investigated metabolite distribution in the oral cavity of healthy volunteers (n = 9). As can be seen from Figures 7 and 8 consistent results for the different locations were observed across the study population. Moreover, location-specific metabolite patterns were identified, underscoring the need for spatially resolved biochemical analysis of the human oral cavity. Finally, we compared our results with a similarly designed study where NMR rather than MS was used for metabolite identification (Meleti et al., 2020). Despite the difference in the number and nature of identified molecules, due to the different instrumentation implemented, comparable individual metabolomic profiles per location were observed including the more dilute fluid content of BT samples when compared with CC. Particularly for head and neck cancer, a spatially resolved metabolomic profiling approach at or around the diseased area allowing the study of malignant tissue in a non-invasive manner might allow one to observe disease-relevant (local) biochemical patterns. Moreover, multi-omics integration as well as correlation with changes in the local microbiome might shed light on metabolite cross talk as well as pathophysiological mechanisms for notoriously hard-to-treat head and neck malignancies. Moreover, inflammatory diseases such as periodontal disease or gingivitis might locally be sampled and biochemically investigated, which considering the local production and metabolism of pro- and anti-inflammatory mediators, might help to better understand physiological phenomena such as inflammation resolution in human subjects. Overall, our here-described spatial sampling protocol will enable human studies biochemically investigating diseases of the oral cavity and also serves as a primer for the local sampling of other relevant bodily fluids and locations, e.g., wound fluid, pus, and tear drops. In conclusion, we systematically evaluated the suitability of several collection devices for spatially resolved sampling and untargeted metabolomics profiling of saliva. We identify the Sugi device as an ideal tool for SRS sampling, presenting the following features: (1) high volume absorption capacity with high reproducibility of sample volume recovery across the different volumes tested; (2) very low level of background contamination, which does not interfere with untargeted metabolomics analysis; and (3) a significant and reproducible recovery of salivary features. Finally, we tested the device in a real-life scenario and show that spatially resolved sampling gives rise to location-specific metabolite distributions in the oral cavity, leading to the possibility of discovering novel biology and enabling to draw a spatial link with oral disease, as well as spatially correlate with other omics, such as transcriptomics, proteomics, and/or glycomics. We are confident that our approach will prove useful in the large-scale investigation of local molecular changes related to diseases of the oral cavity, such as, for example, periodontal disease or head and neck cancer.

### Limitations of study

The here-presented study was aimed at identifying the most suitable collection device for untargeted metabolic profiling of saliva. If the protocol is being used for other bodily fluids, e.g., tears, blister or wound fluid, a brief validation of our protocol for these purposes should be considered. Moreover, we have not investigated the quantitative behavior of specific metabolite classes but rather aimed at a comprehensive evaluation of detection devices. In other words, if the protocol is being applied for the targeted analysis of specific metabolites, a dedicated validation procedure has to be carried out. Moreover, in case our protocol is being used to sample specific disease areas, data normalization should be considered. This can be achieved by different approaches as, for example, total area normalization as well as normalization based on collected area or volume. Ultimately, each study has its own limitations and requirements, and the suitability of our protocol for specific purposes should be investigated on an individual basis.

## STAR★Methods

### Key resources table

**Table:** 

REAGENT or RESOURCE	SOURCE	IDENTIFIER
**Chemicals, peptides, and recombinant proteins**		

Methanol LC-MS grade	Merck	1.06035.2500; CAS: 67-56-1
Water LC-MS grade	Honeywell	14263-2L; CAS: 7732-18-5
Acetonitrile LC-MS grade	Honeywell	34967-2.5L; CAS: 75-05-8
Formic acid additive for LC-MS	Honeywell	56302-10X1ML; CAS: 64-18-6

**Critical commercial assays**		

Tubed sterile Dryswab™	MWE	https://www.mwe.co.uk
Salivette®	Salivette	https://www.sarstedt.com
SalivaBio Oral Swab	Salimetrics	https://salimetrics.com
Sugi® absorbent swab	Questalpha	https://www.questalpha.com
Supelco® Bio-SPME C18 coated fibers	Merck	https://www.merckgroup.com

**Deposited data**		

Raw and analyzed data	This paper	Metabolights: MTBLS2905

**Software and algorithms**		

MS-DIAL (version 4.20)	Tsugawa et al., 2015	http://prime.psc.riken.jp/compms/msdial/main.html
R (version 4.0.3)	(R Core Team, 2020)	https://cran.r-project.org/

**Other**		

BEH HILIC (130Å, 1.7 μm, 2.1 mm × 100 mm)	Acquity UPLC	186003461
BEH HILIC VanGuard (130Å, 1.7 μm, 2.1 mm x 5 mm)	Acquity UPLC	186003980

### Resource availability

#### Lead contact

Further information and requests for resources should be directed to and will be fulfilled by the Lead Contact, M. Giera (m.a.giera@lumc.nl).

#### Materials availability

This study did not generate new unique reagents.

#### Data and code availability

This paper does not report any original code; all codes used are mentioned in the statistical analysis section. The data supporting the current study have been deposited at Metabolights and are publicly available as of the date of publication. Accession number is listed in the key resources table.

### Experimental model and subject details

This study was conducted according to the Declaration of Helsinki principles. The study protocol for the systematic evaluation of collection devices was approved by the Leiden University Medical Center (LUMC) voluntary donors service who has been granted the right for sampling whole saliva from six healthy volunteers by the Ethics Committee and of the board of directors of the LUMC. In addition, a second study protocol has been reviewed and approved by the CCB Science Committee (LUMC) for comparing whole saliva (WS) and spatially resolved saliva (SRS) collected from 10 healthy donors. Informed consent was obtained from all study subjects. Data and samples were encoded (pseudonymized). In accordance with the study inclusion criteria, donors were male of age between 18 and 45 years old. All samples were collected between 8:30 a.m. and 10:30 a.m. and all subjects were requested to avoid any food and liquid intake (except water) from one hour prior sample collection. Among the subjects, subject number two (WS vs. SRS comparison) was excluded from the study because of medication treatment which extensively affected the observed saliva metabolic profile.

### Method details

#### Materials

Methanol was purchased from Merck (Darmstadt, Germany). If not stated otherwise, all chemicals were purchased from Honeywell (Charlotte, NC, USA). All solvents were of LC-MS grade.

In the present systematic evaluation, five collection devices were selected, four swabs and one solid phase micro extraction coated fiber (SPME) device. (**1**) MWE - Tubed sterile Dryswab™ (Dryswab), a cotton bud type, is recommended for general clinical use, forensic use and saliva collection. (**2**) Salivette - salivette® plain cotton swab (Salivette), a stable and biocompatible synthetic cotton fiber roll, which is optimized for the collection of saliva (de Palo et al., 2009; van Faassen et al., 2017). (**3**) Salimetrics – SalivaBio Oral Swab (SOS), a synthetic, chemically inert material, that has been designed for minimal volume retention of saliva and (chemical) analysis (Topkas et al., 2012). (**4**) Questalpha – Sugi® absorbent swab (Sugi), a high-performance cellulose swab with a high tensile strength, ideal for retaining absorbed liquids. (**5**) Merck - Supelco® Bio-SPME C18 coated fibers (SPME), allows for the selective extraction of a broad range of analytes from biological samples while repelling unwanted macromolecules and matrix (Bessonneau et al., 2013, 2015).

#### Sample collection

Donors were recommended to drink 0.5 L of water from three h to one h prior to saliva collection and to refrain from eating, smoking, drinking (except water), and brushing their teeth from one h before collection. Donors were requested to rinse their mouth with plain water ten min before saliva collection. For saliva collection two different methods were used. WS was collected by spitting, SRS was collected with the Sugi device. In case of WS collection, donors were asked to spit in a 50 mL falcon tube for a duration of two min. SRS collection was carried out using the Sugi swab, by pressing the swab on the surface of the oral areas of interest for a duration of 2 min before being stored in a centrifugation storage tube (Salimetrics). The oral locations sampled were below the tongue (BT), above the tongue (AT), right and left cheeks (RC, LC). Samples collected this way were directly stored at −80°C in the case of WS samples or stored at −80°C post swab centrifugation at 1500 ×g for 15 min at 20°C. After centrifugation, the extracted saliva was processed further or stored until analysis.

#### Spatially resolved sampling step by step protocol

Procedure:

1. Ask the donor to rinse his/her month with still water 10 min before saliva collection.

Notes: during the 10 min prior collection the donor should limit talking as much as possible

2. Ask the donor to open his/her month.
3. Press the Sugi swab softly onto the surface of the location of interest.
4. Ask the donor to close his/her mouth.
5. Wait 2 min.

Notes: (1) when placing the swab on the location of interest be careful to not touch other external or oral surfaces (external surface, lips, teeth, etc.), (2) to ensure a proper collection, ask the donor to maintain their head in a neutral position.

6. Pull out the swab and place the Sugi device in a storage tube.

Notes: the swab stick does not fit completely inside the swab storage tube, but it is possible to squeeze the swab inside using the purple cap.

7. Centrifuge the storage tube at 1500 ×g for 15 min at 4°C.
8. Remove the swab from the storage tube after centrifugation.
9. Extracted saliva is ready to be stored or processed further.

Anatomical description of oral locations and tips:

1. Above the tongue consists in the center of the body of the tongue, no root and apex. After having placed the swab on the tongue body ask the donor to gently press his/her tongue against the palate.
2. Below the tongue consists of the floor of the mouth delimited by the inferior arch teeth. After having placed the swab on the mouth floor move the swab stick to the side avoiding the front teeth and allowing the mouth to close. Moreover, ask the donor to press the tongue against the mouth floor and to make sure that both the bottom of the tongue and the mouth floor are in contact with the swab.
3. Cheek consists of the bottom of the cheeks next to the first molar tooth. Once the donor opens his/her mouth pose the swab on the cheek in line with the first molar and brush the swab on the cheek down to the cheek bottom.

Notes: Specific anatomical indications are essential to ensure reproducibility during sampling procedures.

#### Instrumental parameters

Chromatography was performed using a DIONEX UltiMate 3000 system (Thermo Scientific) for all swab evaluation tests and a Nexera X2 (Shimadzu) was employed for the spatially resolved sampling test in human subjects. In both cases, an Acquity UPLC BEH HILIC (130Å, 1.7 μm, 2.1 mm × 100 mm) (Waters) was used. The column was kept at 40°C, and the injection volume was 2 μL. Gradient elution was performed using 10 mM ammonium acetate in water (eluent A), and acetonitrile (eluent B). The flow rate was 0.4 mL min^-1^. The gradient was as follows: 0-1 min, 95% B, 9 - 10 min, 40% B, 10.5 – 17.5 min, 95 % B (equilibration). A maXis Impact HD (Q-TOF instrument, Bruker) was used for detection during all swab evaluation analyses, whereas a TripleTOF 6600 (Q-TOF instrument, Sciex) was used for the spatially resolved sampling test. The mass spectrometer was scanning from *m/z* 100-1000 for all MS1 experiments and, in the case of spatially resolved sampling test, from *m/z* 50-1000 for MS2 experiments. Measurement conditions are described in Table S1 (maXis Impact HD) and Table S2 (TripleTOF 6600).

### Quantification and statistical analysis

Spectra deconvolution and peak alignment were performed using MS-DIAL (version 4.20) (Tsugawa et al., 2015). A detailed description of all applied parameters can be found in Table S3. Peaks were detected when present in at least 100 % of replicates belonging to the same collection device, sample type, aqueous control or reference group. All detected features were validated as follows. (**1**) In order to remove features which were not sample related, a background threshold (BgT) was computed using an aqueous control sample (worked up LC-MS water sample) (*BgT = mean (control_group (peak_area)) + sd (control_group (peak_area)) × 3*) (n = 5), consequently every value equal or below the threshold was defined as missing value (NA), (**2**) features that were not detected in all biological replicates of the reference group were excluded from further analysis (NA > 0). (**3**) In addition, to evaluate the reproducibility of the measurements, a relative standard deviation (RSD) was computed for all features detected in the reference group (*RSD = sd (reference_group (peak_area)) / mean (reference_group (peak_area)) × 100*), and consequently a 20 %cut off was applied and any feature characterized by RSD > 20 % was excluded from further analysis. For the background contamination and spatially resolved sampling test a pool of all samples was used as reference group (n = 5 and n = 13), whereas in the case of molecular features recovery test non-sampled saliva was set as reference group (n = 5). Metabolite identifications were carried out for the spatially resolved sampling only. The computed MSDIAL total score was based on isotope ratio similarity, accurate mass similarity, and MS2 similarity. For all other tests, MS detection was performed at MS1 level only. Considering the fixed time and volume used during the sampling procedure for the systematic evaluation of collection device and the qualitative comparison between collection methods, additional data normalization steps were not applied. Data analyses and visualizations were carried out using R (version 4.0.3) (R Core Team, 2020). The packages used during data analysis were tidyverse (Wickham et al., 2019), broom (Robinson, 2021), gtable (Wickhams and Pedersen, 2019), cowplot (Wilke, 2019), patchwork (Pederson, 2020a), ggforce (Pederson, 2020b), ggVennDiagram (Gao, 2019), ropls (Thévenot et al., 2015) and pheatmap (Kolde, 2019).

#### Volume recovery

To evaluate volume recoveries and reproducibility, absorption and desorption capacities were measured and subsequently the percentage of recovery (Rec %) was calculated. In practice, the absorption capacity (Abs) of all swabs was evaluated by weighing, before and after the absorption of saliva from healthy volunteers. Subsequently, the absorbed water was extracted from each device following the guidelines provided by the manufacturer. Where no protocol was provided (Dryswab and Sugi), the protocol from the SOS swab was applied. The devices were centrifuged at 1500 ×g for 15 min at 20°C using swab storage tubes supplied by Salimetrics apart from Salivette which was centrifuged at 1000 ×g for 2 min at 20°C using a swab storage tube supplied by Salivette. Subsequently, the desorbed amount of water (Des) was determined by weighing. Rec % was determined as follows: *Rec % = Des / Abs × 100 %*. The SPME device was excluded from this investigation. All devices were tested with different initial volumes of tap water (0.2 mL, 0.5 mL, and 1.0 mL) and each volume was tested in quintuplicate.

#### Background contamination

We assessed the level of chemical contamination derived from the devices and its effect on the LC-MS background signal. Each device was exposed to 0.2 mL of LC-MS grade water for 2 min. According to guidelines provided by Supelco®, the SPME device was preconditioned by first immersing the fibers in 200 μL of a 85:15 MeOH:H_2_O (v/v %) for 15 min and subsequently in 200 μL of a 15:85 MeOH:H_2_O (v/v %) solution until samples extraction. Metabolites were recovered by placing the fiber in 200 μL of 85:15 ACN:H_2_O solution and shaking the immersed fiber for 30 min using an orbital shaker. The resulting solution was injected into the LC-MS system. For the other devices, water was extracted as described under the volume recovery section. After centrifugation, samples were transferred to 2 mL Eppendorf tubes, MeOH was added in 4:1 (v/v) ratio and the samples were placed in a freezer at -30°C for 20 min. Subsequently, samples were centrifuged at 18000 ×g for 20 min at 4°C. The supernatant was transferred to 2 mL glass vials. Samples were dried under a gentle stream of nitrogen and reconstituted in 85:15 ACN:H_2_O (v/v %) solution with the same volume of the original samples. Reconstitution was assisted by sonication for 1 min and vortexing for 5 sec. The so obtained aqueous extracts were analysed in both ESI+ and ESI- mode. The results were compared with an aqueous control sample which had undergone identical sample preparation steps without involving the collection device, in the case of SPME, blank injections (85:15 ACN:H_2_O (v/v %)) were used as control. The analysis was carried out in quintuplicate.

#### Molecular features recovery

To approach the “real-life” situation and to evaluate the suitability of a collection device for untargeted analysis, molecular features recovery was assessed by comparing sampled saliva with non-sampled saliva. WS collected from healthy donors was divided into 200 μL aliquots. All devices were exposed to this saliva for 2 min in quintuplicate. The samples were extracted and processed as previously described under the background contamination section. Additionally, 5 aliquots of saliva were processed without being in contact with any device (reference samples). To evaluate collection devices, we computed the percentage of overlap among features recovered, as well as features reproducibility, and fold change of each observed molecular feature between non-sampled saliva (reference samples) and sampled material. Fold changes were calculated referring to the reference samples set at 100 %. Hence, a fold change of 1.0 would indicate a high fidelity between sampled material and untreated samples, whereas a fold change < 1.0 would indicate feature loss and > 1.0 feature enrichment or background contamination. The obtained results were corrected with an aqueous control sample generated by sampling LC-MS water which had undergone identical sample preparation.

#### Spatially resolved sampling

With the aim of testing our protocol, spatially resolved samples were compared with a conventional WS collection method. WS and spatially resolved samples (AT, BT, LC, and RC) were collected from nine male healthy donors. An aliquot of 30 μL of each sample was processed as previously described in background contamination section and analysed in ESI+ mode. The four locations in the oral cavity sampled have been chosen as a test case for the following anatomical reasons: (**1**) the four locations are separated from each other by arch teeth (AT and BT vs. LC & RC) and tongue (AT vs. BT), (**2**) the saliva flux of each location is provided by different combinations of salivary glands: AT – tongue and palate minor glands, BT – submandibular glands, sublingual glands, and tongue minor glands, LC & RC (CC) parotid glands, and cheeks minor glands. Among all spatially resolved samples collected, nine swabs (one AT, one BT, three LC and four RC) presented samples loss (no saliva recovered) and were excluded from further analysis. MS detection was performed using tandem mass spectrometry as described in the Instrumental parameters section and in Table S3. Detected features were matched with the MS library provided by MS-DIAL. Results were corrected using LC-MS water sampled with the Sugi swab. All features matching our quality criteria, as described under the quantification and statistical analysis section, were classified in common and uniquely detected molecular features based on collection methodology and location sampled. Subsequently, spatially resolved samples comparison (AT vs BT vs CC)) was further analysed using multivariate data analysis (PCA and PLSDA) as well as cluster analysis. In both cases, data were centered, UVscaled and log10 transformed prior analysis. The PLSDA model was validated throughout a permutation test, as well as model parameters and visual comparison with the PCA score plot. Finally, non-parametric statistical tests were applied to determine true significance. In the case of sampling type comparison, a Mann-Whitney test was applied, whereas a Kruskal-Wallis test was applied for spatially resolved comparisons. Outcome were visualized as Venn diagrams and heatmaps.

## Supplemental information

Document S1. Figures S1–S6 and Tables S1–S3

Data S1. Identified and detected molecular features, related to Figures 6, 7 and 8 as well as Figure S5 and S6 The table displays all features fulfilling our quality criteria (section 2.4) listing alignment ID, MS library match (metabolite name), *m/z*, retention time, MS2 spectrum, INCHKEY. Metabolite names reported are the library matches with the “highest score” between the list of metabolite candidates per feature. The score is the total score from isotope ratio similarity, accurate mass similarity, and MS2 similarity. When the highest score is below the set threshold, the score is recalculated without MS2 similarity, such features are reported as “w/o MS2:”, which refers to a lack of MS2 spectral data. Ultimately, “Unknown” features failed to satisfy the identification cut-off using the above criteria. Moreover, presence (1) or absence (0) of metabolites is reported for whole saliva (WS) and spatially resolved samples (AT, above the tongue; BT, below the tongue; CC, cheeks). Moreover, p-values are reported for both sampling types (WS vs SRS) and location (AT, BT, and CC) comparison. In the case of comparison for sample types, p-values were computed using Mann-Whitney test, whereas for location comparison Kruskal-Wallis test was used. Finally, VIP values from the PLSDA are also reported.
