# Supplementary material for: Spatially resolved sampling for untargeted metabolomics: A new tool for salivomics
Source: iScience. 2021 Jun 24;24(7):102768. doi: 10.1016/j.isci.2021.102768 (PMC8271151; doi:10.1016/j.isci.2021.102768)
Supplement: Document S1. Figures S1–S6 and Tables S1–S3 [file mmc1.pdf]

iScience, Volume *24*

Supplemental information

Spatially resolved sampling for untargeted metabolomics: A new tool for salivomics

Alessio Ciurli, Maximiliam Liebl, Rico.J.E. Derks, Jacques J.C. Neefjes, and Martin Giera

1. Supplemental information figures

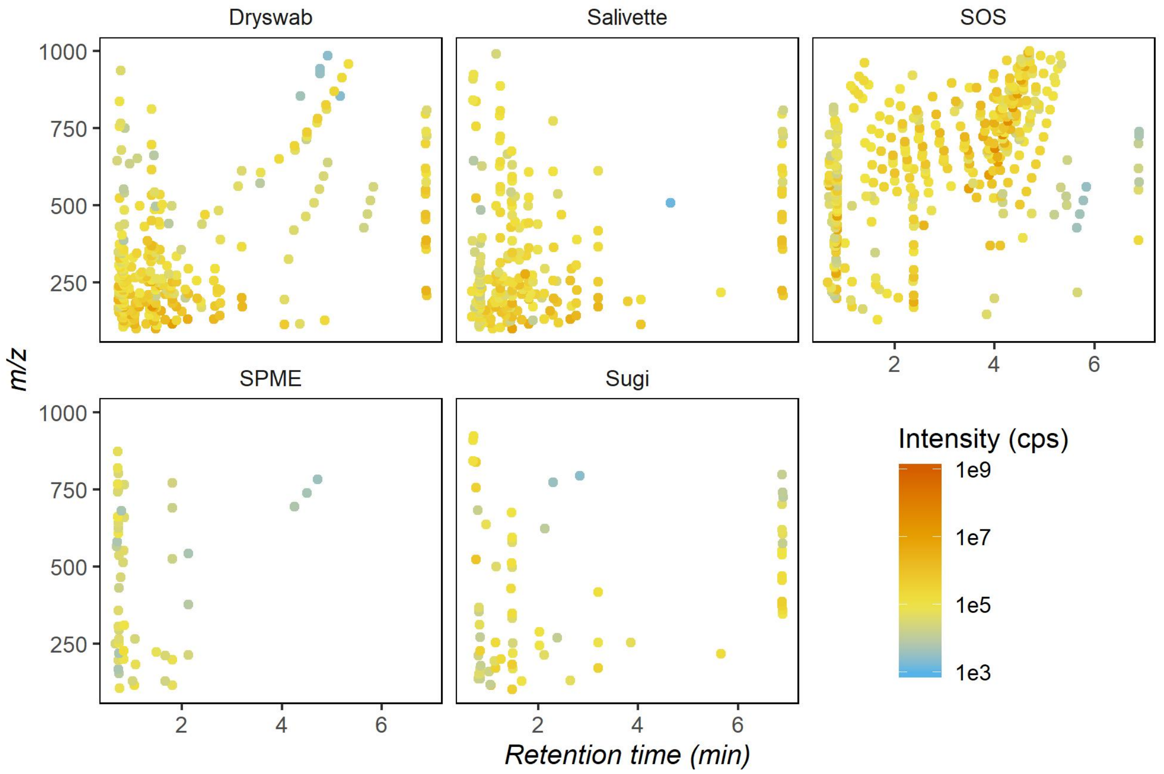

Fig. S1 Background contamination by ESI- mode, related to Figure 2. The sequence of 2d plots displays the contaminants released per collection device. Contaminants are displayed based on their mass to charge (y axis) and retention time (x axis) coordinates and colored based on the average of signal intensities (cps) as shown in the legend. Data are represented as mean (n = 5).

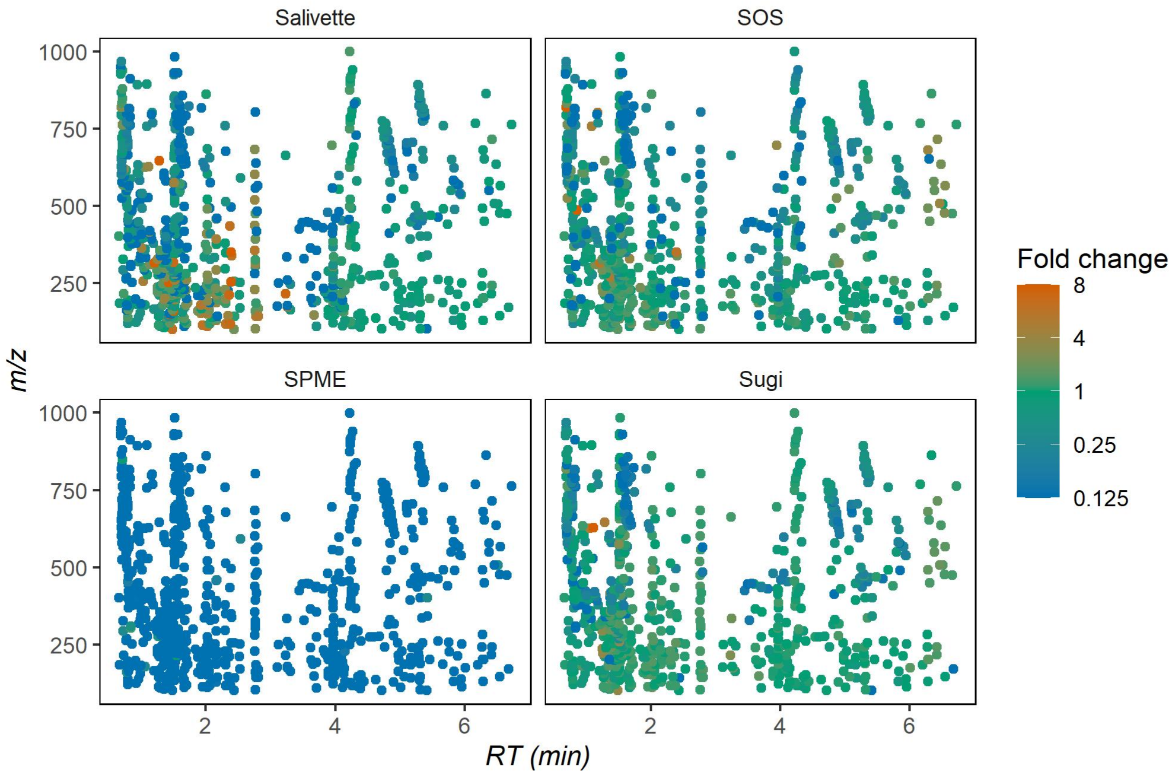

Fig. S2. Molecular features recovery by ESI- mode, related to Figure 3. The sequence of 2d plots displays the molecular features recovered per collection device. Molecular features are displayed based on their mass to charge (y axis) and retention time (x axis) coordinates. Color bar indicates the fold change of the average signal intensity of sampled saliva over the average of non-sampled saliva . Data are represented as mean (n = 5).

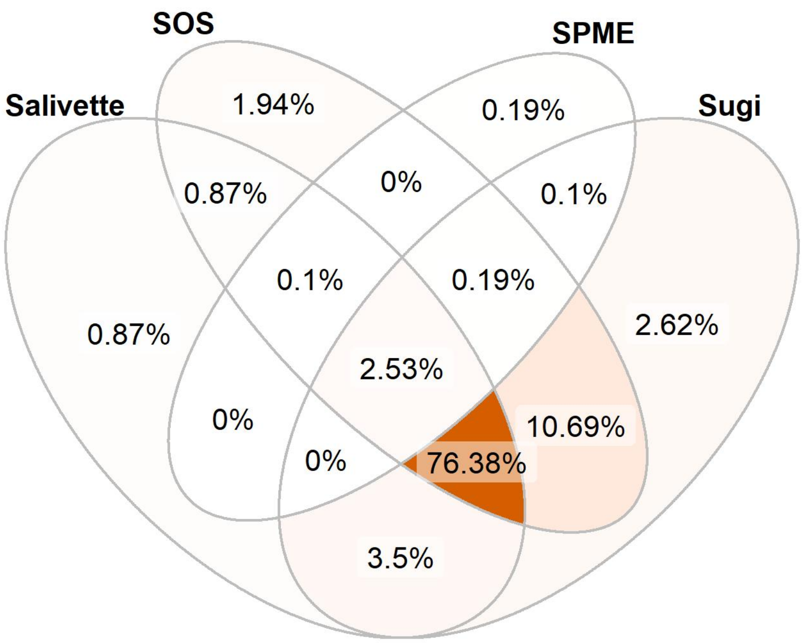

Fig. S3 Overlap of features captured by ESI- mode, related to Figure 4. The Venn diagram displays the percentage of features detected by each device (n = 5) and the overlaps among the devices. Venn diagram sections are colored based on the percentage of features belonging to each section.

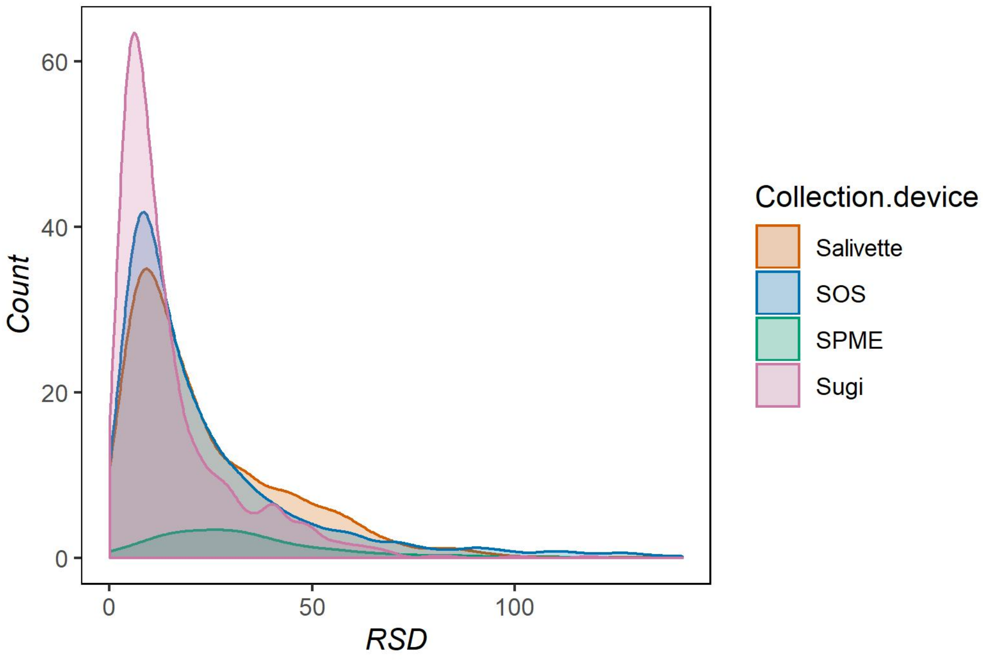

Fig. S4 Reproducibility by ESI- mode, related to Figure 5. The density plot displays the distribution of RSDs belonging to the molecular features recovered by each device (n =5), as reported in the legend.

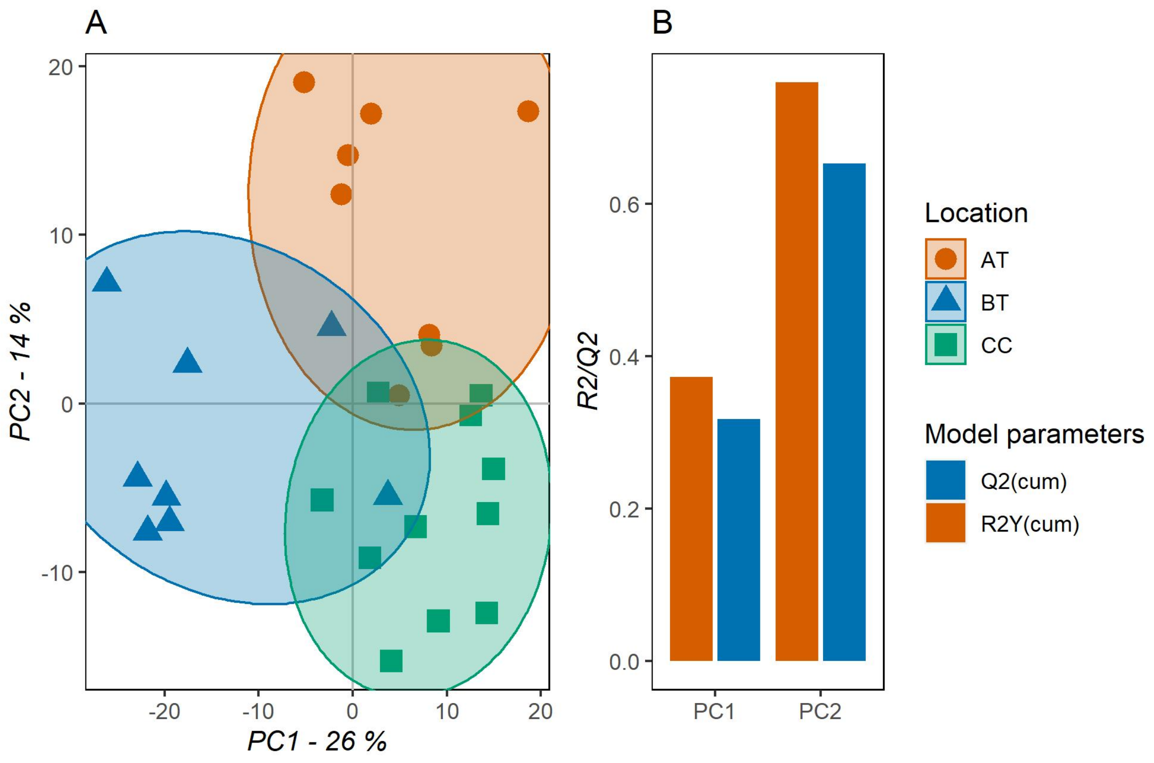

Fig. S5 Diagnostic plots, related to Figure 7. The 2D plots display (A) the score plot from PCA for principal component one (x axis) and two (y axis), on the axis label the explained variance for each principal component is reported. Samples are colored based on the location of collection (AT = above the tongue, BT = below the tongue and CC = cheeks) and (B) the bar plot reporting the cumulative explained variance (R2Y(cum)) and the cumulative predicted variance (Q2(cum)) from PLSDA (ATn = 8, BTn = 8 and CCn = 11).

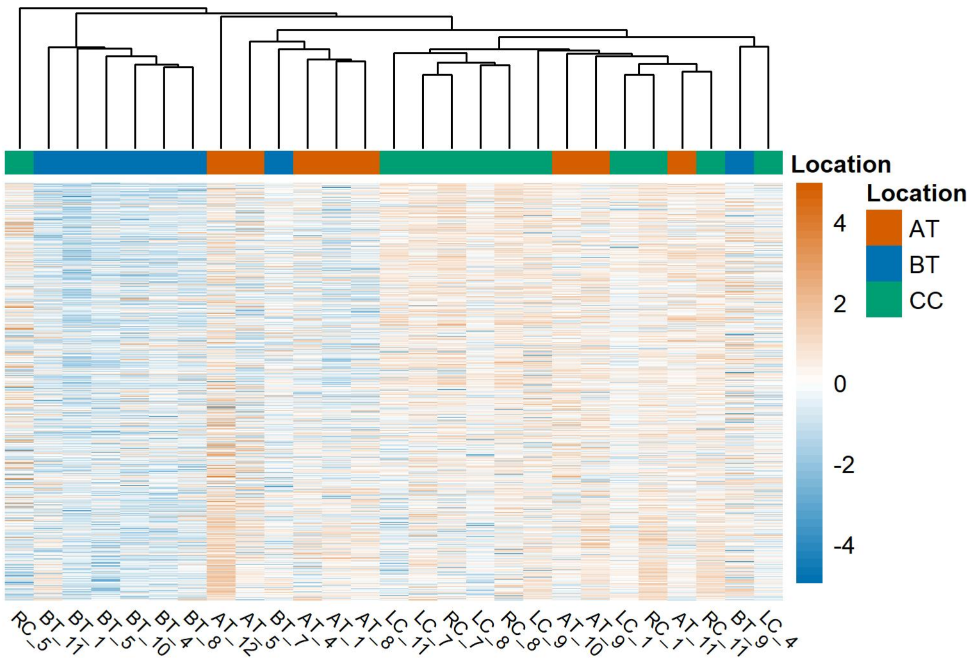

Fig. S6 Heatmap showing all detected features, related to Figure 8. All samples were labelled by location and subject number (x axis labels) and annotated based on the location of collection (AT = above the tongue, BT = below the tongue and CC = cheeks), as reported in the legend. Data are represented as centered, UVscaled and Log10 transformed (ATn = 8, BTn = 8 and CCn = 11).

2. **Supplemental information tables**

Instrument	maXis Impact HD (Q-TOF instrument, BRUKER)
**Source**	
Source Type	ESI
Ion Polarity	Positive
Set Nebulizer	1.6 Bar
Focus	Active
Set Capillary	4500 V (-4000 V)
Set Dry Heater	350°C
Set End Plate Offset	-500 V
Set Dry Gas	6.0 L min^{-1}
Scan Begin	100 *m/z*
Scan End	1000 *m/z*
**Ion Optics**	
Set Funnel 1 RF	200.0 Vpp
Set Funnel 2 RF	300.0 Vpp
**Quadrupole**	
Set Ion Energy (MS only)	5.0 eV (-5.0 eV)
Set Isolation Mass (MS only)	80.00 *m/z*
Collision Energy	10.0 eV (-10.0 eV)
Set Collision Cell RF	300.0 Vpp

Tab S1. Bruker maXis Impact MS method parameters, related to STAR Methods. All parameters used during the MS measurements for all swabs evaluation experiments are reported. When parameters diverge between ESI+ mode and ESI- mode, ESI- parameters were reported in brackets.

Instrument	TripleTOF 6600 (Q-TOF instrument, Sciex)
**Source**	
Source Type	ESI
Ion Polarity	Positive
CUR gas	30
GAS 1	30
GAS 2	25
ISVF	5500 V
TEM	500°C
**General MS**	
Cycle time	0.62 s
DP	80 eV
**TOF MS1**	
CE	10 eV
Start mass	100 Da
End mass	1000 Da
Accumulation time	50 ms
**TOF MS2**	
Acquisition method	IDA
With intensity greater than	100 cps
Switch after	20
Exclude former target ions	Never
Mass tolerance	50 ppm
CE	30 eV
CES	15 eV
Start mass	50 Da
End mass	1000 Da
Accumulation time	26 ms

Tab S2. Sciex Triple TOF 6600 MS method parameters, related to STAR Methods. All parameters used during the MS measurements of healthy volunteers are reported. Parameters are reported for ESI+ mode.

MS-DIAL Version	4.00
**Project**	
MS1 Data type	Centroid
MS2 Data type	Centroid
Ion mode	Positive
Target	Metabolomics
Mode	ddMSMS
**Data collection parameters**	
Retention time begin	0.5
Retention time end	10.5
**Centroid parameters**	
MS1 tolerance	0.01
MS2 tolerance	0.025
Mass range begin	100
Mass range end	1000
**Isotope recognition**	
Maximum charged number	2
**Peak spotting parameters**	
Mass slice width	0.1
**Peak detection parameters**	
Smoothing method Linear	Weighted Moving Average
Smoothing level	2
Minimum peak width	8
Minimum peak height	1000
**Deconvolution parameters***	
Sigma window value	0.5
MS2Dec amplitude cut off	2
Exclude after precursor	True
Keep isotope until	0.5
Keep original precursor isotopes	False

**MSP file and MS/MS identification setting***	
MSP file	MSMS-Public-Pos-VS15
Retention time tolerance	
Accurate mass tolerance (MS1)	0.01
Accurate mass tolerance (MS2)	0.05
Identification score cut off (%)	75
Using retention time for scoring	False
Using retention time for filtering	False
**Text file and post identification (retention time and accurate mass based) setting***	
Retention time tolerance	0.1
Accurate mass tolerance	0.01
**Advanced setting for identification***	
Relative abundance cut off	0
Top candidate report	True
**Adduct ion setting***	
[M+H]+	True
[M+NH4]+	True
[M+Na]+	True
[M+K]+	True
[M+ACN+H]+	True
**Alignment parameters setting**	
Retention time tolerance	0.05
MS1 tolerance	0.015
Retention time factor	0
MS1 factor	0.5
Peak count filter	0
N % detected in at least one group	100
Gap filling by compulsion	False
**Tracking of isotope labels**	
Tracking of isotopic labels	False

**Ion mobility**	
Ion mobility data	False
* Settings were used only in case of the spatially resolved sample comparison, where metabolite identification was performed.	

Tab S3. MS-DIAL settings, related to STAR Methods. All settings used for detection, deconvolution, alignment, and identification are reported.
